# Patient-reported gastrointestinal symptoms in gastric cancer after laparoscopic distal gastrectomy

**DOI:** 10.3389/fonc.2024.1421643

**Published:** 2024-10-30

**Authors:** Shuomeng Xiao, Zhi Ding, Fazhi Zhao, Chao Yang, Ping Zhao, Xiaodong Chen, Xiang Zhou, Huali Zhou, Rui Xu

**Affiliations:** Department of Gastric Surgery, Sichuan Clinical Research Center for Cancer, Sichuan Cancer Hospital & Institute, Sichuan Cancer Center, Affiliated Cancer Hospital of University of Electronic Science and Technology of China, Chengdu, China

**Keywords:** gastric cancer, postoperative gastrointestinal symptoms, laparoscopic distal gastrectomy, Roux-en-Y, Billroth-II with Braun anastomosis

## Abstract

**Purpose:**

This study aimed to compare postoperative gastrointestinal symptoms between patients who underwent laparoscopic distal gastrectomy with Roux-en-Y (R-Y) and Billroth-II with Braun (B-II B) reconstruction.

**Methods:**

This observational study retrospectively analyzed 151 patients (110 in R-Y group and 41 in B-II B group) who underwent laparoscopic distal gastrectomy from January 2020 to December 2021. A comparison was made regarding surgical outcomes, perioperative nutritional and inflammatory markers, postoperative dietary patterns, and gastrointestinal symptoms between the two groups.

**Results:**

The operation time was longer in the R-Y group than the B-II B group (261.00 ± 56.17 min versus 239.88 ± 57.78 min, *p* = 0.046). However, there were no significant differences in the length of hospital stay, ASA classification, complications, nutritional and inflammatory indexes, or recovery of postoperative diet between the two groups. Additionally, there were no significant differences in the occurrence of postoperative gastrointestinal symptoms in the post-discharge week (PDW) 1 and postoperative month (POM) 1 between the B-II B and R-Y groups.

**Conclusions:**

Abdominal distention emerged as the main gastrointestinal symptom burden in patients with gastric cancer undergoing laparoscopic distal gastrectomy. Both Billroth-II with Braun and R-Y reconstructions exhibited a high and similar incidence of gastrointestinal symptoms in the short term. Therefore, medical staff should pay attention to the management of gastrointestinal symptoms in these patients postoperatively.

## Introduction

Gastric cancer is a prevalent gastrointestinal malignancy, ranking as the fifth highest in incidence and fourth highest in mortality globally. In 2020, around one million patients were diagnosed with gastric cancer, leading to the death of 769,000 individuals (1). Surgery remains a critical component in the treatment of gastric cancer, offering the potential to enhance patient outcomes and extend survival rates (2, 3). Laparoscopic surgery has emerged as a popular minimally invasive technique in clinical settings, showcasing benefits such as reduced trauma, lower postoperative complication rates, quicker recovery times (4), and even increase quality of lymphadenectomy (5).

Laparoscopic distal gastrectomy has increasingly gained popularity as a viable alternative to open gastrectomy for patients with stage I-III gastric cancer. Noteworthy studies, such as the Korean Laparoscopic Gastrointestinal Surgery Study (KLASS) group’s KLASS 01 trial, have demonstrated the feasibility and safety of laparoscopic gastrectomy for early gastric cancer (6, 7). Similarly, the Chinese Laparoscopic Gastrointestinal Surgery Study (CLASS) group’s CLASS-01 trial showcased the noninferiority of laparoscopic distal gastrectomy compared to open surgery for locally advanced gastric cancer, emphasizing the comparable 3-year disease-free survival rates between the two approaches (8). Among the various laparoscopic reconstruction methods available, Billroth II and Roux-en-Y reconstructions are commonly utilized in laparoscopic distal gastrectomy due to their ease of operation and anti-reflux properties.

Not only is surgery the standard treatment method, but it also impacts quality of life and triggers postoperative symptoms (9). The presence of postoperative symptoms often triggers anxiety, slows down recovery, and further diminishes quality of life (10–12). The development of postoperative symptoms is influenced by surgical techniques and the type of reconstruction, with limited research on the correlation between laparoscopic reconstruction and postoperative gastrointestinal symptoms. Hence, this study was devised to examine the variances in postoperative gastrointestinal symptoms between patients undergoing laparoscopic distal gastrectomy with R-Y and B-II B reconstruction.

## Methods

### Patients

**Figure 1:**
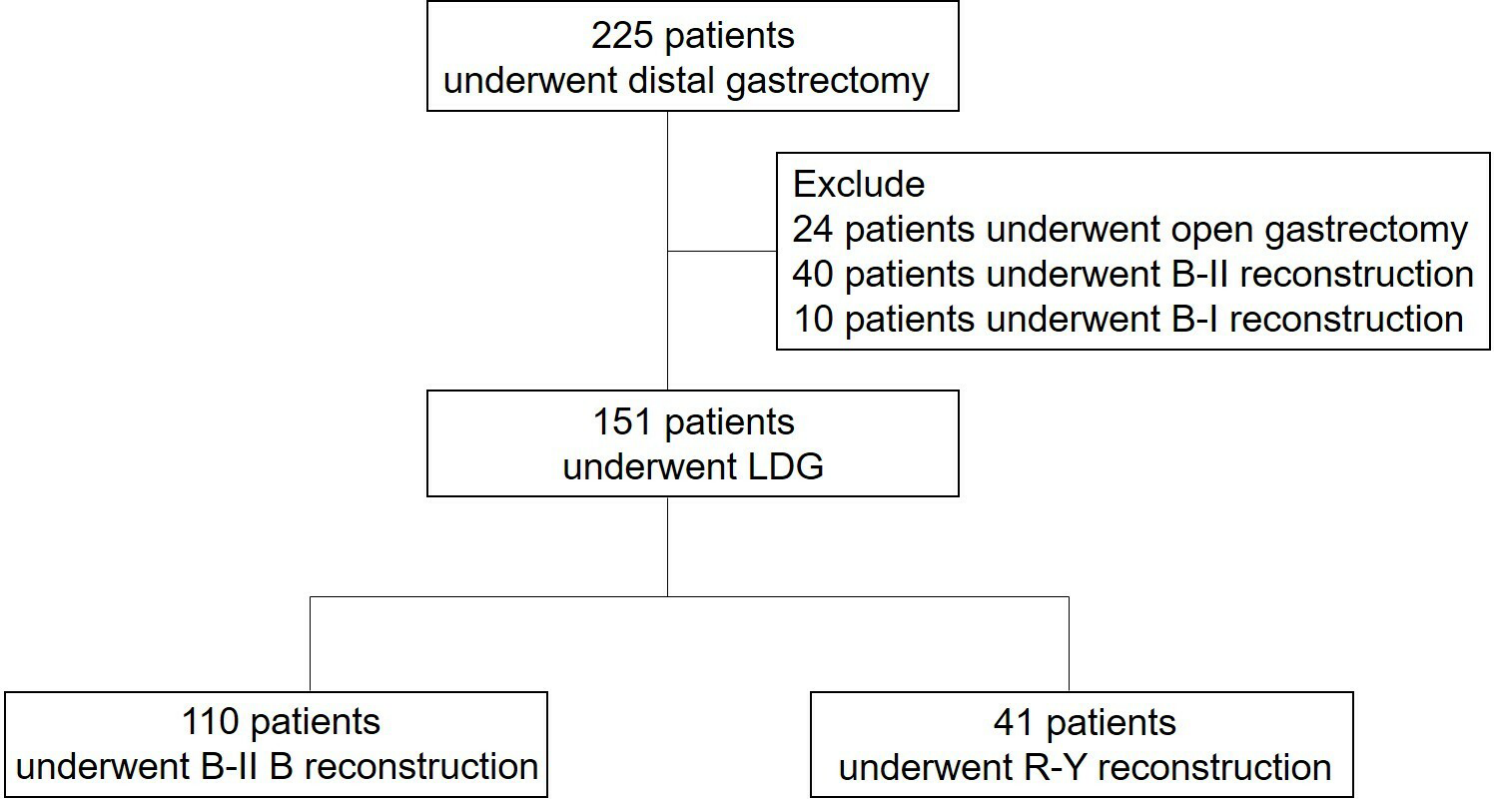
Cohort Chart. B-II B, Billroth-II with Braun anastomosis; R-Y, Roux-en-Y anastomosis.

225 patients diagnosed with gastric cancer and who underwent laparoscopic distal gastrectomy at the Department of Gastric Surgery in Sichuan Cancer Hospital in China from January 2020 to December 2021 were initially included in this observational study. 24 patients who had open gastrectomy, 40 patients with B-II reconstruction, and 10 patients with B-I reconstruction were excluded. Ultimately, the analysis was conducted on a total of 151 patients (**Figure 1**).

The inclusion criteria applied were as follows: (1) age ≥18 years, (2) diagnosed with gastric cancer through biopsy pathology, (3) TNM stage pT1-4NxM0, (4) underwent laparoscopic radical gastrectomy (distal gastrectomy with B-II B or R-Y reconstruction).

The exclusion criteria were as follows: (1) had another malignancy; (2) previous antitumor therapy, such as chemotherapy, radiation, targeted therapy, or immunotherapy; (3) had pulmonary, cardiovascular, or renal disease; (4) had a mental illness; (5) had missing data. Approval for the study was obtained from the Ethics Committee of Sichuan Cancer Hospital, and informed consent was collected from each patient in accordance with the Declaration of Helsinki.

### Type of reconstruction

All patients underwent laparoscopic radical gastrectomy for gastric cancer according to the fifth edition of the Japanese gastric cancer treatment guidelines of the Japanese Gastric Cancer Association (13). The type of reconstruction was selected by the surgeon according to intraoperative factors and his preference.

#### B-II B anastomosis

A gastrojejunostomy was created in an end-to-side fashion approximately 40 cm below the ligament of Treitz, utilizing the anterocolic pathway. Following this, a Braun anastomosis was performed roughly 20 cm below the initial gastrojejunostomy. The entry points of the stapler at the gastrojejunostomy and jejunojejunal anastomosis were sealed with Barbed suture. Closure of Petersen’s defect and the mesenteric defect was done using polydioxanone 3/0 sutures.

#### R-Y anastomosis

After dividing the jejunum 15 cm from the ligament of Treitz, it was brought through the anterocolic route. The distance between the gastrojejunostomy and the jejunojejunal anastomosis measured around 40 cm. Closure of all stapler entry points was done using barbed suture. Additionally, both Petersen’s defect and the mesenteric defect were closed with polydioxanone 3/0 sutures.

### Data collection

#### Clinicopathological characteristics

Clinical data, including age, gender, body mass index (BMI), PG-SGA score, TNM stage, weight, ASA classification, operating time, length of hospital stay, and postoperative complications were collected from the electronic medical records system. The nutritional assessment of patients before surgery was evaluated using the Patient-Generated Subjective Global Assessment (PG-SGA).

#### Hematological index

Peripheral blood was collected preoperative and on the day of discharge. The nutritional indexes included prealbumin (PAB), hemoglobin (HB), and lymphocyte (LYM) counts. The inflammatory indexes included neutrophils (NEUT), platelets (PLT), C-reactive protein (CRP) levels.

#### Patient reported PGISs and diet

Patients reported on postoperative gastrointestinal symptoms (PGISs) and diet. A healthcare provider trained in qualitative interviewing techniques conducted individual interviews with each patient. These interviews occurred during the first postoperative discharge week (PDW) and first postoperative month (POM). Each interview lasted approximately 20 minutes per patient. PGISs consisted of abdominal distention, pain, diarrhea, vomiting and dysphagia. The diet included a liquid diet, a semiLiquid diet, a soft diet, and a general diet. Participants were asked about their PGISs and diet during the interview.

#### Statistical analysis

SPSS 20.0 software (IBM Corp.) was utilized for conducting statistical analysis. Mean ± standard deviation was used to present quantitative variables such as age, BMI, operating time, length of hospital stay, PG-SGA score, weight, and hematological index. Frequencies and percentages were employed for categorical variables. Quantitative data were examined using the t-test, whereas categorical data were analyzed using either the chi-square test or Fisher test. Statistical significance was considered at a two-sided p value of <0.05.

## Results

### Patient characteristics

The present study analyzed 151 individuals diagnosed with gastric cancer who underwent laparoscopic distal gastrectomy with B-II B or R-Y reconstruction. Of the participants, 102 (67.5%) were classified as TNM stage I-II, while 49 (32.5%) were classified as TNM stage III. Within the B-II B group of 110 patients, there were 73 males and 37 females, with a mean age of 57.65 ± 11.36 years. The characteristics of the selected patients were detailed in **Table 1**, which showed no significant differences in age, gender, BMI, PG-SGA score, and TNM stage between the two groups.

**Table 1:** Baseline patient factors.

Characterisitic	B-II B group (n=110)	R-Y group (n=41)	*P value*
Age (years)	57.65 ± 11.36	58.10 ± 10.13	0.827
Gender			0.823
Male	73 (66.4)	28 (68.3)	
Female	37 (33.6)	13 (31.7)	
BMI (kg/m²)	23.13 ± 3.39	23.23 ± 3.20	0.867
PG-SGA score	5.65 ± 4.49	6.29 ± 4.11	0.422
T stage			0.261
T1	41 (37.3)	10 (24.4)	
T2	21 (19.1)	10 (24.4)	
T3	24 (21.8)	14 (34.1)	
T4	24 (21.8)	7 (17.1)	
N stage			0.786
N0	58 (52.7)	19 (46.3)	
N1	16 (14.5)	5 (12.2)	
N2	20 (18.2)	10 (24.4)	
N3	16 (14.5)	7 (17.1)	
TNM stage			0.508
I-II	76 (69.1)	26 (63.4)	
III	34 (30.9)	15 (36.6)	

B-II B, Billroth-II with Braun anastomosis; R-Y, Roux-en-Y anastomosis; BMI, body mass index; PG-SGA, patient-generated subjective global assessment.

### Comparison of surgical Indicators between the two groups

The operation time for the B-II B group was significantly shorter than that of the R-Y group (239.88 ± 57.78 min vs 261.00 ± 56.17 min, p=0.046). Additionally, the lengths of hospital stay for the two groups were similar, with the B-II B group at 9.45 ± 2.79 days and the R-Y group at 9.83 ± 3.95 days. Both groups had minimal postoperative complications, with the B-II B group experiencing one case of pneumonia and one case of abdominal infection, while the R-Y group had only one case of lymphorrhagia. Overall, there were no significant differences in the length of hospital stay or the incidence of postoperative complications between the two surgical groups (**Table 2**).

**Table 2:** Comparison of surgical outcomes between two groups of patients.

Variable	B-II B group (n=110)	R-Y group (n=41)	*P value*
ASA classification			0.588
I	20 (18.2)	10 (24.4)	
II	81 (73.6)	29 (70.7)	
III	9 (8.2)	2 (4.9)	
Operating time (min)	239.88 ± 57.78	261.00 ± 56.17	0.046
Hospital stays (d)	9.45 ± 2.79	9.83 ± 3.95	0.570
Postoperative complications	2 (1.8)	1 (2.4)	1.000
Pneumonia	1	0	
Abdominal infection	1	0	
Lymphorrhagia	0	1	
Leakage	0	0	

B-II B, Billroth-II with Braun anastomosis; R-Y, Roux-en-Y anastomosis.

### Comparison of nutritional and inflammatory indexes between the two groups

There were no statistically significant differences between the two groups in the preoperative and discharge nutritional indexes (PAB, HB, LYM, weight) and inflammatory indexes (NEUT, PLT, CRP) (p>0.05). However, the nutritional indexes at discharge were lower than those preoperatively, and the inflammatory indexes were higher than those preoperatively, as presented in **Table 3**.

**Table 3:** Comparison of nutritional and inflammatory indexes between two groups of patients.

Variable	Time	B-II B group (n=110)	R-Y group (n=41)	*P value*
PAB (mg/L)	Preoperation	217.07 ± 52.50	204.79 ± 62.37	0.227
	Discharge	138.29 ± 47.16	128.00 ± 56.46	0.265
HB (g/L)	Preoperation	126.18 ± 22.80	121.24± 23.84	0.244
	Discharge	107.21 ± 21.88	107.54 ± 20.12	0.934
NEUT (10^9^/L)	Preoperation	3.42 ± 1.41	3.26 ± 1.31	0.527
	Discharge	4.99 ± 2.22	5.17 ± 2.21	0.658
PLT (10^9^/L)	Preoperation	197.82 ± 65.15	194.73 ± 69.97	0.800
	Discharge	230.83 ± 74.20	246.88 ± 120.63	0.429
LYM (10^9^/L)	Preoperation	2.49 ± 9.69	2.23 ± 4.58	0.872
	Discharge	1.68 ± 2.40	1.65 ± 2.44	0.964
CRP (mg/L)	Preoperation	2.62 ± 5.70	4.40 ± 12.19	0.225
	Discharge	37.52 ± 35.37	39.30 ± 43.87	0.798
Weight (kg)	Preoperation	60.46 ± 10.97	59.95 ± 10.13	0.795
	Discharge	58.68 ± 10.48	57.70 ± 9.11	0.595

B-II B, Billroth-II with Braun anastomosis; R-Y, Roux-en-Y anastomosis; PAB, prealbumin; HB, hemoglobin; NEUT, neutrophils; PLT, platelets; LYM, lymphocyte; CRP, C-reactive protein.

### Comparison of patient reported diet and PGISs between the two groups

**Figure 2:**
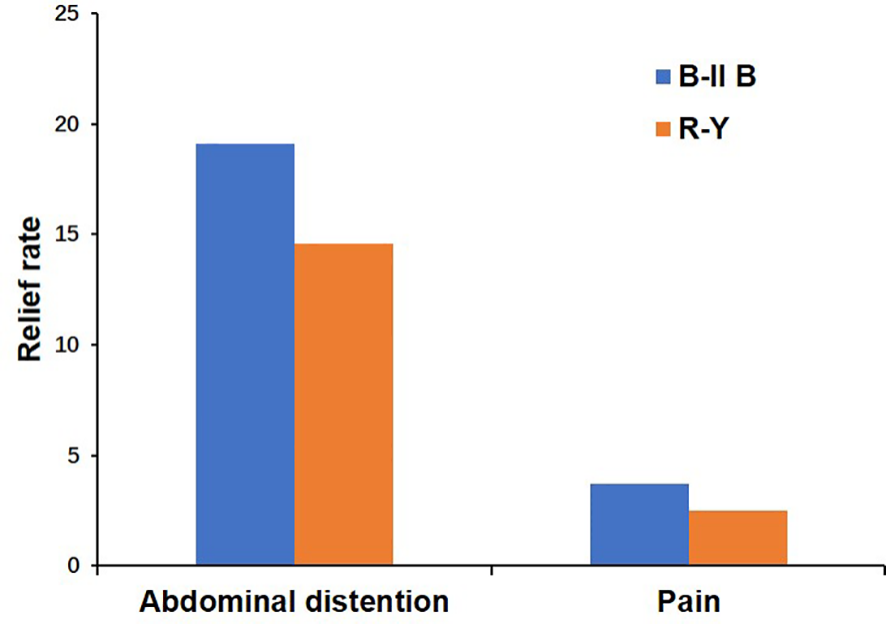
The relief rates of abdominal distention and pain between the B-II B and R-Y groups from PDW 1 to POM 1. B-II B, Billroth-II with Braun anastomosis; R-Y, Roux-en-Y anastomosis; PDW, postdischarge week; POM, postoperative month.

During PDW 1 and POM 1, there were no statistically significant differences in postoperative dietary status (*p*>0.05) (**Table 4**). In PDW 1, a majority of patients (80, 72.7%) reported PGISs in the B-II B group, comprising abdominal distention (53, 48.2%), pain (18, 16.4%), vomiting (5, 4.5%), diarrhea (4, 3.6%), and dysphagia (0, 0%). Similarly, in the R-Y group, a significant number of patients (25, 61%) reported PGISs, primarily abdominal distention (18, 43.9%), pain (4, 9.8%), vomiting (1, 2.4%), diarrhea (1, 2.4%), and dysphagia 1, 2.4%). In POM 1, approximately half of the patients (56, 50.9%) reported PGISs in the B-II B group, comprising abdominal distention (32, 29.1%), pain (14, 12.7%), vomiting (5, 4.5%), diarrhea (4, 3.6%), and dysphagia (1, 0.9%); Similarly, in the R-Y group, 18 (43.9%) patients reported PGISs, with abdominal distention (12,29.3%), pain (3, 7.3%), vomiting (1, 2.4%), diarrhea (1, 2.4%), and dysphagia 1, 2.4%) being the most common symptoms. However, there were no significant differences in PGISs between the two groups at the two time points (*p*>0.05) (**Table 5**). The B-II B group had greater rates of abdominal distention and pain relief than did the R-Y group during the period from PDW 1 to POM 1(19.1% *vs* 14.6%, and 3.7% *vs* 2.5%, respectively), but these differences were not statistically significant (**Figure 2**).

**Table 4:** Comparison of recovery of postoperative diet between two groups of patients.

Variable	Time	B-II B group (n=110)	R-Y group (n=41)	*P value*
Diet				
Liquid diet	PDW 1	2 (1.80)	0 (0)	–
	POM 1	1 (0.9)	0 (0)	–
Semiliquid Diet	PDW 1	82 (74.5)	27 (65.9)	0.289
	POM 1	4 (3.6)	1 (2.4)	1.000
Soft diet	PDW 1	26(23.6)	14 (34.1)	0.193
	POM 1	93 (84.5)	37 (90.2)	0.368
General diet	PDW 1	0 (0)	0 (0)	–
	POM 1	12 (10.9)	3 (7.3)	0.761

B-II B, Billroth-II with Braun anastomosis; R-Y, Roux-en-Y anastomosis; PDW, postdischarge week; POM, postoperative month.

**Table 5:** Comparison of postoperative gastrointestinal symptoms between two groups of patients.

Variable	Time	B-II B group (n=110)	R-Y group (n=41)	*P value*
Abdominal distention	PDW 1	53 (48.2)	18 (43.9)	0.639
	POM 1	32 (29.1)	12 (29.3)	0.983
Pain	PDW 1	18 (16.4)	4 (9.8)	0.306
	POM 1	14 (12.7)	3 (7.3)	0.563
Diarrhea	PDW 1	4 (3.6)	1 (2.4)	1.000
	POM 1	4 (3.6)	1 (2.4)	1.000
Vomiting	PDW 1	5 (4.5)	1 (2.4)	1.000
	POM 1	5 (4.5)	1 (2.4)	1.000
Dysphagia	PDW 1	0 (0)	1 (2.4)	NA
	POM 1	1 (0.9)	1 (2.4)	0.471

B-II B, Billroth-II with Braun anastomosis; R-Y, Roux-en-Y anastomosis; PDW, postdischarge week; POM, postoperative month.

## Discussion

In fact, LDG has been widely implicated in gastric cancer in the clinic. With the publication of the CLASS 01 results, LDG has become a preferred surgical method for treating gastric cancer. However, the selection of reconstructive type after LDG is a problem among surgeons.

Billroth I, Billroth II, and Roux-en-Y anastomosis are commonly used for reconstruction after LDG, but they are still not perfect (14, 15). Billroth I anastomosis is limited due to its anastomotic tension and relatively high recurrence risk, while Billroth II anastomosis is limited because of the high rate of bile reflux (16, 17). Thus, B-II B reconstruction and R-Y reconstruction have been widely used due to their good operability and antireflux effect in LDG (18). Yalikun et al. found that the B-IIB group had a shorter operative duration compared to the R-Y group (19). However, a meta-analysis revealed that there was no significant difference in hospital stay or complications between the two groups (20). This suggests that while the B-IIB procedure may be quicker, it does not necessarily lead to better outcomes in terms of postoperative recovery and complication rates. In our study, we also found that the B-II B surgical procedure was simpler and faster than the R-Y group (239.88 ± 57.78 min versus 261.00 ± 56.17 min, *P*=0.046), and the rates of postoperative complications were not significantly different between the B-II B group and the R-Y group (*P*>0.05). Technically, the keys to saving time are avoidance of jejunal mesenteric division and a wider space (21). This result clearly showed that the B-II B anastomosis method was more efficient and safer.

Patients who undergo surgery may experience an inflammatory reaction, which is a natural response of the body (22). A severe inflammatory response can lead to tissue and organ damage, which can seriously threaten patients. The results of our study indicated that the discharge NEUT, PLT and CRP levels were higher in the B-II B and R-Y groups than before surgery, but no significant differences were observed between the two groups at the two time points, which was the same as the results of Chi. et al. (23) This indicated that the inflammatory response was stimulated after gastrectomy, and mild inflammation remained present on the day of discharge. Gastric cancer surgery can affect the digestive and absorption functions of the gastrointestinal tract, which affects the patient’s nutritional status. Lee. et al. showed that nutritional indexes decreased after gastrectomy; for example, after undergoing gastrectomy, patients typically experience an average relative body weight loss of 2.5% of their preoperative body weight (24). In our study, we found that the discharged HB, LYM indexes and weight were lower in the B-II B and R-Y groups than before surgery, but no significant differences were observed between the two groups at the two time points. Malnutrition is associated with increased morbidity and mortality, prolonged hospital stays, increased complication rates, and decrease survival rates (25). Thus, it is crucial to monitor the nutritional status of patients following surgery and offer intervention when necessary.

An ideal procedure for gastrointestinal reconstruction aims to reduce postoperative complications and the rate of PGIS, accelerate postoperative recovery and improve quality of life. Various studies have showed that PGIS frequently occurred after gastrointestinal surgery (26, 27). In this study, the prevalence of PGISs was 58.3% in PDW 1 and 48.3% in POM 1 after laparoscopic distal gastrectomy. Even with minimally invasive procedures, the incidence of PGISs was still more than half, but gradually decreased over time. Additionally, we found that abdominal distention was the main symptom in the two groups. Abdominal distention is often associated with gastrointestinal motility disorders. Theoretically, Interstitial cells of Cajal (ICC) receive inputs from motor neurons as well as mechanical stimuli, and in turn, they produce and transmit electrical rhythmic patterns to regulate the motility of the gastrointestinal system (28–30). Several studies have shown that the damage to motility caused by gastrointestinal resection and inflammation is due to injury to the ICC. Ding et al. reported that fewer ICCs in the submucosa of the small intestine and a greater inflammatory response in the muscularis mucosae of the small intestine were found in the R-Y group after gastrectomy (31). This suggests that the surgical procedure may have an impact on the distribution of ICCs and the level of inflammation in the small intestine. Based on the KLASS-07 database, Park et al. found that B-II B could reduce STO22 reflux symptoms compared with R-Y (32). Moreover, Xie et al. showed that Uncut R-Y with high recanalization rate even aggravated gastrointestinal symptoms (33). In this study, compared with R-Y reconstruction, B-II B reconstruction tended to relieve abdominal distention faster, which may be related to gastrointestinal continuity and a relatively lower inflammatory response, but the observed difference was not statistically significant, potentially due to the limited number of patients underwent R-Y reconstruction. Thus, we considered that expanding the sample size of R-Y reconstruction might help to highlight the advantages of B-II B reconstruction.

In this study, several limitations were identified. First, this was a single-center and retrospective study, thus which may limit the generalizability of the results to a larger population. Second, an uncertain choice of reconstruction could lead to selection bias, which might influence the outcomes of this study. Finally, we only observed postoperative gastrointestinal symptoms and excluded other reported symptoms, which might have introduced bias. Further research needs to be conducted.

## Conclusions

In summary, abdominal distention is the main gastrointestinal symptom burden in patients with gastric cancer who underwent laparoscopic distal gastrectomy. In the short term, Billroth-II with Braun and R-Y reconstruction still have a high and similar incidence of gastrointestinal symptoms, and deserves the attention of medical staff.

## Data Availability

The raw data supporting the conclusions of this article will be made available by the authors, without undue reservation.
